# Analysis of the N-glycosylation profiles of the spike proteins from the Alpha, Beta, Gamma, and Delta variants of SARS-CoV-2

**DOI:** 10.1007/s00216-023-04771-y

**Published:** 2023-06-24

**Authors:** Dongxia Wang, Jakub Baudys, Sarah H. Osman, John R. Barr

**Affiliations:** Division of Laboratory Sciences, National Center for Environmental Health, Centers for Disease Control and Prevention, Atlanta, GA USA

**Keywords:** N-Glycosylation, SARS-CoV-2 spike variants, Mass spectrometry

## Abstract

N-Glycosylation plays an important role in the structure and function of membrane and secreted proteins. Viral proteins used in cell entry are often extensively glycosylated to assist in protein folding, provide stability, and shield the virus from immune recognition by its host (described as a “glycan shield”). The SARS-CoV-2 spike protein (S) is a prime example, having 22 potential sites of N-glycosylation per protein protomer, as predicted from the primary sequence. In this report, we conducted mass spectrometric analysis of the N-glycosylation profiles of recombinant spike proteins derived from four common SARS-CoV-2 variants classified as Variant of Concern, including Alpha, Beta, Gamma, and Delta along with D614G variant spike as a control. Our data reveal that the amino acid substitutions and deletions between variants impact the abundance and type of glycans on glycosylation sites of the spike protein. Some of the N-glycosylation sequons in S show differences between SARS-CoV-2 variants in the distribution of glycan forms. In comparison with our previously reported site-specific glycan analysis on the S-D614G and its ancestral protein, glycan types on later variants showed high similarity on the site-specific glycan content to S-D614G. Additionally, we applied multiple digestion methods on each sample, and confirmed the results for individual glycosylation sites from different experiment conditions to improve the identification and quantification of glycopeptides. Detailed site-specific glycan analysis of a wide variety of SARS-CoV-2 variants provides useful information toward the understanding of the role of protein glycosylation on viral protein structure and function and development of effective vaccines and therapeutics.

**Supplementary Information:**

The online version contains supplementary material available at 10.1007/s00216-023-04771-y.

## Introduction

The coronavirus disease 2019 (COVID-19) caused by the severe acute respiratory syndrome coronavirus 2 (SARS-CoV-2) emerged in late 2019 and has continuously mutated to form many viral variants [1–3]. The transmembrane spike protein (S) of SARS-CoV-2 mediates viral entry into human cells and is the main target for neutralizing antibodies [4]. This protein is anchored on the virus surface membrane as a homotrimer prefusion structure and each of the protomers consists of two subunits, S1 and S2 [5, 6]. The S1 subunit contains an N-terminal domain (NTD) and a receptor binding domain (RBD) that binds to host cell surface receptor angiotensin-converting enzyme 2 (ACE2). The S2 subunit is comprised of heptad repeats, a fusion peptide, and a transmembrane domain responsible for viral fusion to the host cell membrane [7–10]. Based on the dynamic positions of RBD versus the other parts of the protein body, S can adopt closed (RBD down) and open (RBD up) conformations [5, 7, 10]. The open conformation with one or more RBDs turning upward allows effective binding to human ACE2 receptor while the closed conformation with all three RBDs down is more compact and protected from neutralizing antibodies but does not bind to the ACE2 receptor [5]*.*

Since the outbreak of COVID-19, thousands of mutations have been reported [11]. SARS-CoV-2 virus has evolved into many variants due to mutations mainly on S during multiple waves of COVID-19 infection. There are 9–11 substitutions or deletions occurring on the S protein of four primary variants of concern (VOC) including B.1.1.7 (Alpha), B.1.351 (Beta), P.1 (B.1.1.28/Gamma), and B.1.617.2 (Delta) following the emergence of D614G (Table 1) [12]. The most recently reported Omicron variant has about 40 mutations on the S protein, many more than the average seen in all previously dominant variants [13]. These variants display enhanced infectivity and immune evasion presumably due to the mutations in the spike glycoproteins [14–16].

**Table 1:** Substitutions in the spike protein of SARS-CoV-2 variants^a^

Position^b^	18	19	20	26	69	70	80	138	142	145	156	157	158	190	215	242	243	244	417	452	478	484	501	570	655	681	701	716	950	982	1027	1118	1176
D614G	L	T	T	P	H	V	D	D	G	Y	E	F	R	R	D	L	A	L	K	L	T	E	N	A	H	P	A	T	D	S	T	D	V
Alpha					Del	Del				Del													Y	D		H		I		A		H	
Beta							A								G	Del	Del	Del	N			K	Y				V						
Gamma	F		N	S				Y						S					T			K	Y		Y						I		F
Delta		R							D		G	Del	Del							R	K					R			N				

^a^Letters represent amino acid (single-letter symbol) substitutions of residues in the D614G spike protein sequence. *Del* represents deletion of a residue

^b^Amino acid positions in the D614G spike protein sequence

Protein glycosylation is one of the most common and diverse post-translational modifications (PTM) [17, 18]. N-glycosylation is the result of attaching carbohydrates to asparagine residues within a specific sequon (Asn-X-Ser/Thr, where X represents any amino acid except proline). In contrast, O-glycosylation is the result of attaching mucin-type glycans on serine or threonine residues. Unlike other PTMs, such as protein phosphorylation with a single modifier attached to a few phosphorylation sites, N- and O-glycosylation of proteins possesses the properties of microheterogeneity (multiple glycoforms attached to a particular glycosylation site) and macroheterogeneity (variable glycosylation site occupancy). The functional roles of glycosylation of viral proteins include facilitating proper folding and stability of protein structure, camouflaging immunogenic epitopes, and promoting immune evasion [16, 19–22].

The SARS-CoV-2 S protein is heavily glycosylated containing 22 consensus N-glycosylation sites per protomer [5, 23, 24]. In addition to maintaining protein structure and covering potential epitope sites, other roles of the N-glycans on this protein have been revealed. Molecular dynamic simulations suggest some N-glycans may promote or stabilize the RBD open conformation [25, 26]. Mutation studies demonstrate that glycosylation deletions on both N331 and N343 dramatically reduce pseudotyped virus entry [21]. In vitro and in vivo studies reveal that removal of N-glycans from virions or interfering with host N-glycosylation via inhibitors or drugs results in reduced virion production or infection spread [27–30]. The roles of glycosylation in SARS-CoV-2 have been well reviewed in multiple papers [31–36].

Advanced mass spectrometry (MS) techniques have greatly facilitated the analysis of N- and O-glycoforms of SARS-CoV-2 S proteins by directly characterizing glycan-containing peptides derived from proteins [6, 23, 24, 37–56]. The glycosylation profiles of S have been determined mostly with various constructs of recombinant proteins, including 2- (or 6-) proline stabilized ectodomain, and separate subunits of S1, S2, and RBD, expressed in human, monkey, or insect cells. Glycosylation of the S proteins on SARS-CoV-2 virions has also been characterized. These studies have been summarized well in a recent review article [57]. All 22 N-glycosylation sequons have been found to be occupied by glycans. O-glycoforms have been detected at a few sites but with relative low occupancy [23, 40]. The S ectodomain is predominantly modified by highly processed complex carbohydrates with some oligomannose structures in recombinant samples and native viral proteins.

To investigate whether evolved SARS-CoV-2 variants of concern alter their glycosylation profile, we conducted a mass spectrometric analysis of the N-glycosylation profiles of recombinant S proteins derived from four SARS-CoV-2 VOCs, including Alpha, Beta, Gamma, and Delta, along with the D614G variant as a control. These results provide useful information for the study of SARS-CoV-2 viral proteins and for the development of effective COVID-19 vaccines and therapeutics. While the 22 N-glycan sequons (N17, N61, N74, N122, N149, N165, N234, N282, N331, N343, N603, N616, N657 in S1 subunit; and N709, N717, N801, N1074, N1098, N1134, N1158, N1173, N1194 in S2 subunit) are highly conserved in SARS-CoV-2 variants, primary sequences suggest the formation of two novel potential N-glycosylation sites, N20 and N188, in Gamma S and the elimination of N17 in Delta S. We observed glycosylation at these new Gamma sites in this study.

## Experimental

### Materials

All chemicals were obtained from Sigma–Aldrich (St. Louis, MO) unless indicated otherwise. Endoproteases used in this study, including trypsin (Cat#XV528X, Mass Spectrometry Grade), chymotrypsin (Cat#V1061, Sequencing Grade), Lys-C (Cat#VA1170, Mass Spectrometry Grade), and Asp-N (Cat#V162A, Sequencing Grade), were obtained from Promega (Madison, WI), and alpha-lytic protease (Cat#P8113S, Proteomics Grade) was purchased from New England BioLabs (Ipswich, MA). The recombinant poly-histidine tagged, trimeric ectodomains of the SARS-CoV-2 S variants (D614G, Alpha, Beta, Gamma, and Delta) expressed in HEK293 cells were purchased from R&D Systems (Minneapolis, MN). The sequences of the two S proteins included a mutated furin cleavage site and K986P, V987P (2P) substitutions.

### Digestion of spike proteins

Digestion of full-length ectodomain S protein was performed similarly to previously published methods with some modifications [38]. In short, 1.5–2 µg of full-length S protein ectodomain was denatured and reduced by heating at 60 °C in a 20 mL solution containing 50 mM ammonium bicarbonate (pH 7.9), 0.05% RapiGest SF surfactant (Waters Corporation), and 5 mM DTT. The samples were then alkylated using 15 mM iodoacetamide for 30 min in the dark on a MixMate mixer at 600 RPM. Single enzyme digestions (trypsin, Lys-C, chymotrypsin, alpha-lytic protease, or Asp-N) occurred in triplicate overnight at 37 °C at an enzyme to protein ratio of 1:10 (w/w). For sequential double enzyme digestions, the first digest was performed for 1 h at 52 °C with the first enzyme (trypsin, Lys-C, or Asp-N, 1:10 w/w enzyme:protein) for a quick and effective digestion [37, 38] and the second digest was performed at 37 °C overnight using the second enzyme (chymotrypsin, alpha lytic protease, or Asp-N). The digestions were conducted under identical conditions for different combinations of two enzymes. The reactions were then quenched by bringing the pH of the solution below 3 using 10% formic acid and incubating for 30 min to precipitate unreacted RapiGest. After, the solutions (26 mL) were centrifuged at 4000 RCF for 10 min and transferred to new vials. Each analysis was conducted in technical triplicates with three batches of protein obtained from identical biological preparation.

### Mass spectrometry (MS) analysis

Nanoflow liquid chromatography coupled to electrospray ionization tandem-mass spectrometry (LC–MS/MS) analysis was performed on an Orbitrap Eclipse Tribrid mass spectrometer and an UltiMate™ 3000 RSLCnano chromatography system (Thermo Scientific) using a signature ion triggered EThcD method as described previously [37, 38]. Briefly, 8 µL of each sample (~ 0.5 mg protein) was injected on an integrated trapping column Acclaim PepMap 100, 75 µm × 2 cm length with 100% mobile phase A (0.1% formic acid in water) for trapping and separation was on an EASY-Spray C18 analytical column (Thermo Scientific, Cat#ES904, 75 µm i.d. × 15 cm length, 2 µm 100 Å particles) with gradient elution with mobile B (80% acetonitrile/20% water). The loading column was operated at 5 mL/min for the first 8 min of the analytical run, to perform sample trapping and desalting, then at 8 min the column oven compartment valve was switched to the alternate position to allow analytical flow through the loading column. The elution gradient was 4% B (0.0–8.0 min); 4–10% B (8.0–10.0 min); 10–35% B (10.0–43.0 min); 35–60% B (43.0–46.05 min); 60–95% B (46.05–46.1 min); 95% B (46.1–53.0 min); 95–4% B (53.0–53.1 min); 4% B (53.1–63.0 min) with a flow rate was 300 nL/min.

Mass spectrometry detection was accomplished using a signature ion triggered EThcD method. Precursor scans were acquired by the orbitrap at a resolution of 120,000 (measured at m/z 200), from m/z 375 to 2000. Following MS1 spectrum acquisition, data-dependent MS2 spectra were acquired over a 3-s cycle time, with each spectrum selecting for monoisotopic precursor peptide ions, with a 1.2 m/z isolation window, with charge states between 2 + and 8 + , an intensity threshold of 3.0 e4, and not repeating ions within 15 s of a prior selection. Selected ions were fragmented using HCD with 28% normalized collision energy and centroided data was acquired in the Orbitrap with a standard AGC target, automated maximum injection time, a scan range of 120–2000 m/z, and resolution 30,000. A follow-up MS2 scan using the same selected precursor ion was performed if at least one of three oxonium ions, HexNac (204.0867), HexNac fragment (138.0545), and HexNacHex (366.1396), was detected in the HCD spectrum within 15 ppm mass tolerance. The secondary ion injection used EThcD for fragmentation, with charge-dependent ETD fragmentation and supplemental HCD fragmentation at 35% collision energy. Profile data was acquired in the Orbitrap with 500% normalized AGC target, 150 ms maximum injection time, a scan range of 150–2000 m/z, and a resolution of 50,000.

### Data analysis

MS/MS data were processed using PMi-Byonic (Protein Metrics, version 3.7) node within Proteome Discover (Thermo Scientific, version 2.4) as previously described [38]. Briefly, Byonic searches were performed at cleavage sites specific to each endopeptidase using a semi specific (slow) digestion specificity with 3 allowed missed cleavages. Precursor mass tolerance was set to 6 ppm. Fragmentation type was set to both HCD & EThcD, with fragment mass tolerance set to 20 ppm for HCD and 20 ppm for EThcD. No recalibration was applied. A fixed carbamidomethyl modification on Cys (C) residues and up to two variable modifications per peptide, including deamidation on Asn(N) or Gln (Q) and oxidation on Met (M), were searched. Using the glycan database provided by PMI with the Byonic software, we utilized the N-glycan 182 human no multiple fucose glycan database with the potential of up to two N-glycan sites per peptide. An automatic score cut was applied for the peptide output and a 1% protein FDR or 20 reverse count was assigned. A Byonic™ score of 150 was set as a filter for the identification of glycopeptides. The protein database includes the sequences of all spike variants and common contaminants. Relative abundance of each type of glycans at each site was calculated as the normalized peak intensity ratio of the peptides bearing a particular glycan type over the total glycopeptides. The glycan abundance was represented as the mean of three replicates along with standard deviation of the mean (smaller than 30%). The glycan abundances of some sites were summed from the average values of two peptides bearing the same glycosylation site (Table 2).

**Table 2:** Sum of the relative peak intensities of the highest (first peptide sequence) and second highest (second peptide sequence) abundance peptide groups, each group including peptides with the same peptide sequence but with different N-glycans^a^

Site	Protease(s)^b^	D614G		Alpha		Beta		Gamma		Delta	
		1st peptide	2nd peptide^c^	1st peptide	2nd peptide	1st peptide	2nd peptide	1st peptide	2nd peptide	1st peptide	2nd peptide
N17	Lys-C	99.6%		99.6%		99.5%					
N20	Lys-C/CHTR							58.9%	34.0%		
N61	Lys-C/aLP	100.0%		100.0%		100.0%		100.0%		100.0%	
N74	Asp-N/CHTR	86.4%		93.7%		87.0%		85.9%		81.1%	
N122	Lys-C/CHTR	75.6%	22.3%	56.4%	35.6%	52.1%	38.9%	53.7%	37.6%	55.0%	37.4%
N165	Lys-C	76.7%	12.0%	80.4%		73.2%	10.1%	78.9%	6.6%	83.7%	
N188	Lys-C							99.6%			
N234	Lys-C/CHTR	66.1%	17.2%	89.1%		85.6%		84.9%		86.9%	
N282	Lys-C/CHTR	83.0%		82.5%		84.3%		85.5%		85.0%	
N331	Lys-C/CHTR	48.1%	45.6%	69.2%	22.0%	66.6%	22.6%	62.5%	27.7%	64.3%	25.5%
N343	Lys-C/aLP	94.6%		93.6%		94.5%		91.4%		97.6%	
N603	Lys-C/CHTR	76.7%	23.3%	74.3%	25.7%	78.6%	25.0%	76.1%	23.9%	64.0%	17.4%
N616	Lys-C/CHTR	72.2%	16.3%	71.1%	20.7%	70.1%	21.2%	71.1%	21.7%	73.3%	19.5%
N657	Lys-C/CHTR	100.0%		100.0%		100.0%		100.0%		100.0%	
N709	Lys-C/aLP	100.0%		100.0%		100.0%		100.0%		100.0%	
N717	Lys-C/aLP	100.0%		100.0%		85.3%		100.0%			
N801	Lys-C/aLP	51.9%	22.2%	52.1%	22.3%	65.3%	11.0%	72.0%	4.4%	70.6%	4.0%
N1074	Lys-C	99.1%		99.1%		99.4%		99.6%		99.9%	
N1098	Lys-C/CHTR	79.4%	10.4%	74.3%	15.3%	76.8%	13.1%	76.5%	14.4%	77.2%	14.5%
N1134	Asp-N/CHTR	100.0%		100.0%		100.0%		100.0%		100.0%	
N1158	Asp-N/CHTR, aLP for Delta	80.7%		85.1%		87.0%		80.0%		93.6%	
N1173	Lys-C/aLP, trypsin/Asp-N for Delta	100.0%		100.0%		100.0%		100.0%		78.9%	21.1%
N1194	Lys-C	100.0%		100.0%		100.0%		100.0%		100.0%	

^a^Each value was calculated by dividing the sum of the intensities of target glycopeptides by the total intensity of all peptides containing a specific glycosylation site. The glycosylation on N149, N17 in Gamma and N717 in Delta was not determined due to lack of valid data

^b^In sequential enzyme digestion, for example, Lys-C/CHTR, the first (Lys-C) and second (CHTR) digestions were conducted at 52 °C/1 h and 37 °C/overnight, respectively. *CHTR* represents chymotrypsin; *aLP* represents alpha lytic protease

^c^The amino acid sequence of the 2nd peptide form of a glycosylation site (if any) overlaps with most of the region of the 1st peptide, but differs by 1–3 residues at the N-terminal or C-terminal end

## Results and discussion

### Detection of glycopeptides from recombinant spike proteins

N-glycosylation profiles of five 2-proline-stabilized (2P), recombinant ectodomain S proteins including Alpha, Beta, Gamma, and Delta variants along with S—D614G as a control were characterized by liquid chromatography-tandem mass spectrometry (LC–MS/MS). To provide the best possible variant-specific comparison of N-glycosylation and avoid potential discrepancies caused by different manufacturers and preparation conditions, all recombinant ectodomain (ECD) S proteins used in this study were obtained from one manufacturer (R&D Systems) assuming that the proteins were expressed and purified under the same experimental conditions.

To produce glycopeptides with single sequon and obtain accurate quantitative results, we employed a multiple digestion strategy in this study using several single or dual proteases with different hydrolysis specificities, including trypsin, Lys-C, alpha-lytic protease, trypsin + Asp-N, Lys-C + chymotrypsin, Asp-N + chymotrypsin, and Lys-C + alpha-lytic protease.. In addition, enzyme specificity was set to “semi-specific” during data processing to include those peptides with one nonspecifically cleaved terminus. Several principles were used in selecting valid glycopeptides for each N-glycan site. First, when the sum of the relative intensities of a set of glycopeptides within the same site, same peptide sequence, and different glycans under one hydrolysis condition that exceeded 80% of the total peptides containing the site, this dataset was considered valid for further comparison. If two or more datasets from the different digestions met this criterion, the group with more glycans was selected. Second, if no such dataset was available from all digestions, the second group of the peptides with a sequence slightly different from the first one was considered and the summed abundance of the two groups greater than 80% was selected. Third, the result was confirmed by similar trends in the distribution of glycan forms and types between at least two datasets obtained under different digestion conditions, and the set with a higher number of glycans was selected as the final result reported here (Table 2, Table S1).

N-glycans were detected on all 22 conventional glycosylation sites (sequons) from the glycopeptides with single glycosylation sites with a few exceptions. The glycosylation site N17 in Delta was lost due to the T19R mutation, N17 in Gamma had unresolved abundance from the peptides containing both N17 and N20 (Gamma is the only variant with an N20 glycosylation site), and other research groups have demonstrated that no glycans were detected at N17 of Gamma S protein [55, 56]. In addition, N717 in Delta and N149 in all variants had insufficient MS/MS spectral quality for quantification. In fact, glycopeptides containing N149 or N717 were detected from some digestion experiments, but were not considered for quantitative analysis (data not shown) for the following reasons: (1) the number of detected glycopeptides (only a few) was significantly lower than that of glycopeptides with other sequons; (2) the glycans were present in peptides containing two N-glycosylation site; (3) the glycans were present in more than two peptide forms (amino acid sequence), with no glycopeptides in single or two combined peptide forms having a major abundance (more than 80%). Undetectable or unquantifiable glycosylated occupancy at some sites has also been observed in previous studies [38, 47, 54]. Moreover, the N-glycosylation profiles of two novel sequons, including N20 and N188 caused by the substitution of T20N and R190S, respectively, in Gamma S, were identified.

The typical “bottom-up” mass spectrometry/proteomics approach utilizes proteases such as trypsin with specific cleavage sites to digest a protein into peptides identified via downstream LC–MS/MS analysis. This approach, however, inevitably generates some issues such as nonspecific hydrolysis, incomplete digestion, and/or missed cleavages, resulting in the generation of non-uniform digestion products. The effectiveness of enzymatic digestion of glycoproteins could also be impacted by structural variations and steric hindrance of attached glycans [58]. These typically are not serious issues for discovery applications to identify proteins or posttranslational modifications, but may be problematic for protein quantification, especially for the direct measurement and quantification of glycopeptides for glycosylation profiling. Different sequences of peptides bearing the same glycosylation site affects the ionization and measured abundance of peptides and some long peptides may become undetectable by a typical LC–MS/MS method. These problems might be some of the factors contributing to the inconsistent results of glycosylation analysis of SARS-CoV-2 spike proteins by different laboratories [41, 57]. To overcome these issues and to obtain single sequon containing glycopeptides, digestion with multiple enzymes in single (only one enzyme) or sequential mode (two enzymes) has been widely used in the analysis of glycoproteins and in almost all reports for the analysis of glycosylation of SARS-CoV-2 spike proteins [6, 10, 23, 24, 37, 38, 40–42, 44, 47, 50–55, 59].

As listed in Table 2, we observed that different digestion conditions using single (only one enzyme) or double enzymes were required to obtain reproducible data for various N-glycosylation sites. No single condition alone could produce good results for all sites. In addition, for some sites, such as N1158 and N1173, one condition was optimal for four of the S variants, but the analysis of Delta S proteins required a different protease(s). Interestingly, Lys-C, but not trypsin (the most used enzyme in proteomic analysis of proteins including SARS-CoV-2 S proteins), alone or in combination with one of the other proteases, appeared to be the most efficient and useful enzyme for characterizing most of the N-glycosylation sites in the present study (Table 2). These results highlight the importance of optimizing digestion conditions to facilitate the characterization of glycoproteins. In other words, optimization should focus not only on specific proteins but also the individual glycosylation sites.

It would be ideal to generate glycopeptides with the same amino acid sequence to accurately analyze the glycan forms at any sites. However, it has been reported that glycans at the same site might be present in glycopeptides of different sequences due to nonspecific or missing cleavage [41], which was seen in the present study. The N-glycosylation profiles of 14 of the 23 sites were determined using uniform glycopeptides (one peptide sequence for a specific site). The others, however, were quantified from the glycopeptides of two major glycopeptide sequences (Table 2, Table S1). For instance, some glycan modified N122 resided on the peptide IVN**N**ATNVVIK (amino acid position: 119–129) with a total peptide peak intensity of 75.6% while some peptides containing the N122 site (22.3%) had the sequence LIVN**N**ATNVVIK (118–129), which is one amino acid longer than the former. In addition, some glycans were detected in the former but not in the latter peptide form and vice versa. Omission of the peptides carrying the second sequence could alter the abundance values of various glycoforms at this site. The presence of some non-negligible glycopeptides with glycans bearing on the second sequence demonstrated that the exclusion of such glycopeptides could adversely affect the quantitative result of glycan abundance at specific glycosylation sites, which might also contribute to the inconsistent results reported by different laboratories.

### Distribution of N-glycan type (processing state) on spike variants

**Fig. 1:**
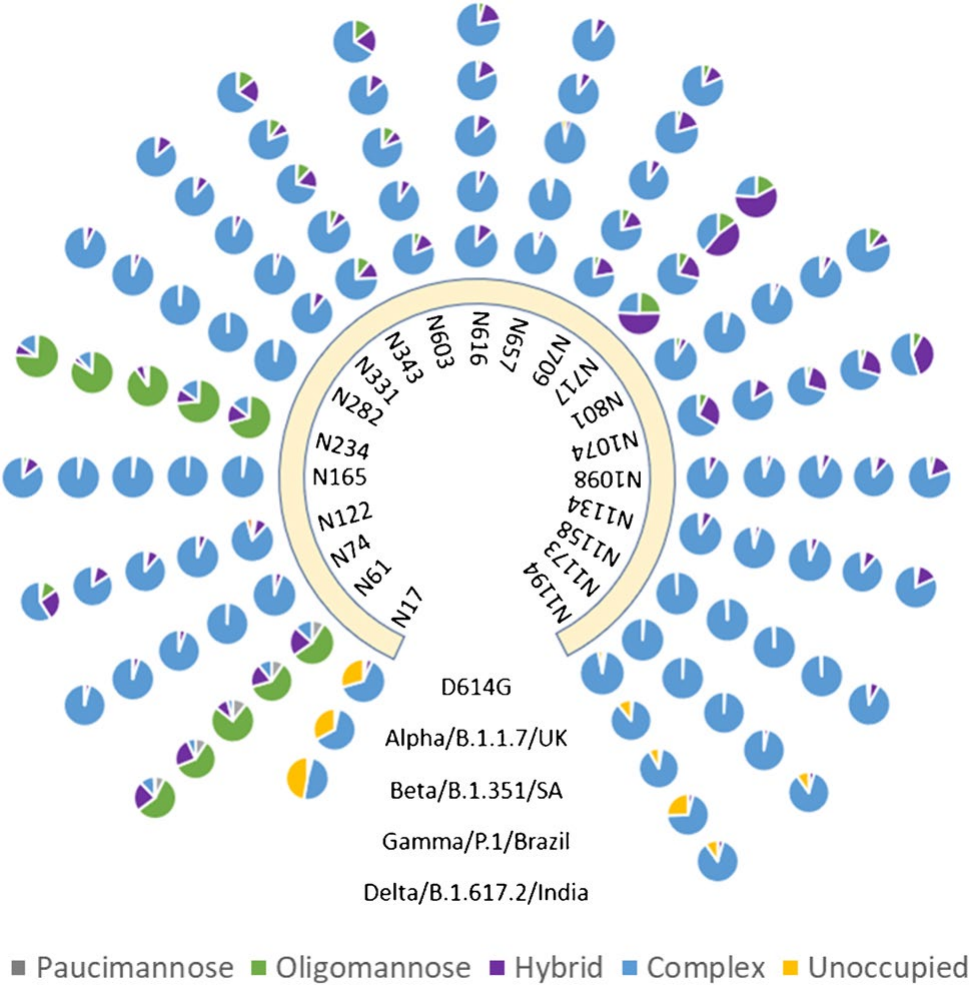
Relative composition of different types of N-glycoforms in ectodomain S proteins of five SARS-CoV-2 variants. The quantitative results of N149, N17 in Gamma and Delta, and N717 in Delta were not available due to either low-quality spectra or missed sequon at N17 upon a specific substitution (Table 1). Glycans are grouped into different types based on their compoisition: (gray) paucimannose (N_1-2_H_0-4_), (green) oligomannose (N_2_H_5-12_F_0-1_A_0-4_), (purple) hybrid (N_3_H_3-6_F_0-1_A_0-4_),(blue) complex (N_4-9_H_3-10_F_0-1_A_0-4_),and (orange) unoccupied. N, HexNAc; H, Hex; F, Fuc; A, NeuAc

The types of the N-glycans were classified in this study as *paucimannose* (1–2 HexNAc and less than 4 Hex groups), *oligomannose* (2 HexNAc and greater than 4 Hex attached), *hybrid* (3 HexNAc and various Hex, Fuc, and NeuAc), and *complex* (4 HexNAc and various Hex, Fuc, and NeuAc). The relative abundance of different types of N-glycans occupied on S proteins of the five variants is depicted in Fig. 1. Our data demonstrate that the majority of 21 determined conventional N-glycosylation sites, except N61, N234, and N717, were occupied by highly processed complex N-glycans with very high occupancy (greater than 80%) in all tested variants, in agreement with the observation on N-glycosylation of spike of early strains of SARS-CoV-2 virus in previous research [23, 51].

Regarding microheterogeneity, the variation in the occupancy of complex N-glycans at individual sites among five S VOCs, high similarity was observed (Fig. 1, Table 3). The differences between the highest and the lowest complex glycan occupancy in all five S proteins were relatively low on some sequons including N17 (15%), N74 (5%), N165 (14%), N282(6%), N331(9%), N616 (15%), N657 (9%), N709 (12%), N801 (15%), N1098 (14%), N1134 (12%), N1158 (8%), and N1173 (14%) while other sites showed low variation in among three or four (but not all five) S variants, such as N122, N343, N603, N616, N1074, and N1194. Interestingly, the Delta S displayed lower abundance of the complex glycans than other variants at many of the sites. For example, only 59% of the site N122 was modified by complex glycans on Delta but other S proteins had 84–93% complex occupancy, suggesting that less dense N-glycans may be sufficient to shield the Delta S for immune evasion or aid viral entry. These results suggest that the overall glycosylation pattern on SARS-CoV-2 S protein has not been significantly altered during viral evolution for D614G through Delta. Our previous study showed considerable changes in glycan content in nearly half of the sequons in the head of S between the original wildtype (D614) and the first major variant D614G [38]. The amino acid substitutions or deletions in the spike variants post-D614G might not affect the formation of matured N-glycans at most N-glycosylation sites. This may indicate that the potential of glycosylation-mediated immune evasion has not been fully utilized by the SARS-CoV-2 virus [55].

**Table 3:** Relative abundance percentage ranges of complex glycans at individual glycosylation sites of the five spike proteins

Site	D614G	Alpha	Beta	Gamma	Delta
N17	63.6%	63.5%	48.7%		
N61	13.2%	10.8%	4.1%	6.8%	11.3%
N74	93.8%	99.1%	94.6%	94.9%	95.8%
N122	84.1%	93.0%	88.2%	84.2%	58.7%
N165	97.3%	98.8%	97.7%	96.8%	85.2%
N234	14.4%	15.2%	2.9%	11.9%	15.3%
N282	97.2%	98.9%	98.3%	93.6%	92.5%
N331	89.3%	95.2%	93.4%	88.3%	86.0%
N343	75.7%	85.2%	71.6%	80.8%	66.1%
N603	81.3%	90.3%	78.6%	86.3%	66.2%
N616	86.9%	92.6%	90.5%	81.9%	77.9%
N657	92.7%	94.9%	93.5%	89.6%	86.3%
N709	77.9%	78.1%	90.0%	79.5%	81.5%
N717	24.4%	71.4%	38.9%	24.0%	
N801	90.9%	96.1%	93.7%	91.1%	80.8%
N1074	65.9%	83.3%	69.9%	69.9%	54.5%
N1098	91.4%	92.7%	90.7%	86.7%	78.9%
N1134	89.7%	93.4%	92.2%	87.7%	81.6%
N1158	99.3%	98.9%	99.2%	99.2%	90.9%
N1173	98.7%	99.4%	99.1%	96.6%	85.9%
N1194	94.2%	87.7%	89.3%	70.3%	66.3%

The N-glycosylation of the S protein of some VOCs has been investigated by several laboratories. Kuo et al. studied the Alpha S proteins, respectively [54]. During the preparation of this manuscript, Newby et al. reported the analysis of N-glycosylation of spike proteins derived from Wuhan-hu-1 and Beta, Gamma, Delta, and Omicron variants [55]. Shajahan et al. described in a preprint the glycosylation mapping of the S proteins from the VOCs of Alpha, Beta, Gamma, Delta, and Omicron [56]. These studies, including ours, consistently demonstrate that processed complex glycans predominantly occupied many of the sequons in the SARS-CoV-2 S protein across different VOCs and there are limited changes in their N-glycosylation profiles (Table S2). On the other hand, discrepancies appeared on some results obtained from these four studies although all these studies used the HEK293 expressed recombinant S proteins. For example, the Kuo group reported a considerably more processed N-glycans at the N122 site of Alpha (63% complex) compared to D614G (31% complex) [54]. However, this conversion is not observed in other studies, probably due to difference in the source of proteins, sample acquisition, data processing, or combination of factors [57]. The results reported by Newby and Kuo’s group show that the N603, N709, N717, and N801 sites of all S variants are predominantly occupied by oligomannose, but complex is the main glycan type revealed by Shajahan and our laboratory. Not surprisingly, our results are highly consistent with those of Shajahan et al. in terms of glycan type distribution, probably because both laboratories use S proteins from the same manufacturer (R&D System). As discussed earlier, more efforts should be made to identify the real sources of discrepancies in glycosylation profiling of glycoprotein by LC–MS/MS or proteomic techniques [57].

Glycosylated N234 is suggested by molecular dynamics (MD) simulation analysis to be an important residue that plays a role in modulating RBD conformation, thereby mediating spike binding to the host receptor ACE2 [25]. It has been determined that N234 is predominantly or 100% occupied by oligomannose in recombinant spike ectodomain trimer [23, 24, 37, 39, 42, 44, 52, 53, 55, 56]. In contrast, complex glycoforms are present in recombinant S1 subunits [37, 42, 44, 52] which suggests that the underprocessed glycoforms at N234 in trimer might be attributed to the quaternary spike structure. Our data revealed that the oligomannose glycoforms at N234 were preserved in the spikes of all studied VOC strains in agreement with the results of other groups [54–56].

N61 is a sequon located at the N-terminal region of the NTD of the S protein. Our data demonstrated that majority of the glycans at this site were oligomannose for five S variants. This is consistent to the observation of Shajahan and Kuo’s group but different from that of Newby’s study where the site is modified mainly by complex type glycans (Table S2) [54–56]. Nonetheless, N234 and N61 are the only two sites occupied by oligomannose across five S proteins in this study, as well in Shajahan’s report with a few exceptions. A recent publication by Munoz-Basagoiti et al. described the interaction between an oligomannose-specific lectin, Cyanovirin-N (CV-N), and SARS-CoV-2 spike protein [60]. They found that the lectin can bind to oligosaccharides on spike protein and block the infection by this virus. Through analysis by isothermal titration calorimetry, gel electrophoresis, western blot, and mass spectrometry techniques, they reveal that the binding stoichiometry is two CV-N molecules to one protomer of spike protein and narrow the potential binding sites down to two of three oligomannose N-glycosylation sites including N61, N122, and N234. Our determination of two oligomannose occupied sites, N234 and N61, correlates well with these findings.

### The profiles of individual N-glycans on potential glycosylation sites

**Fig. 2:**
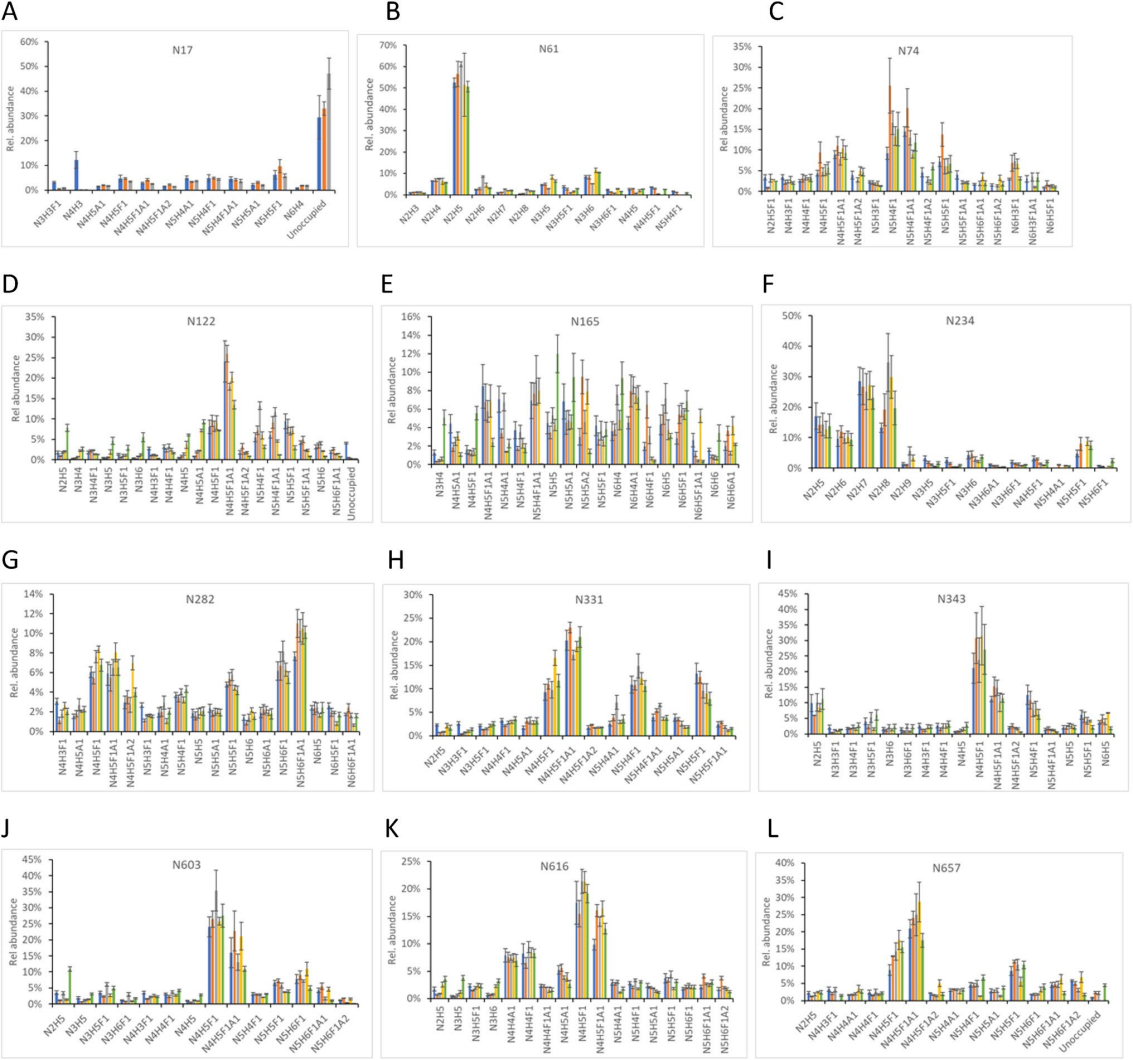

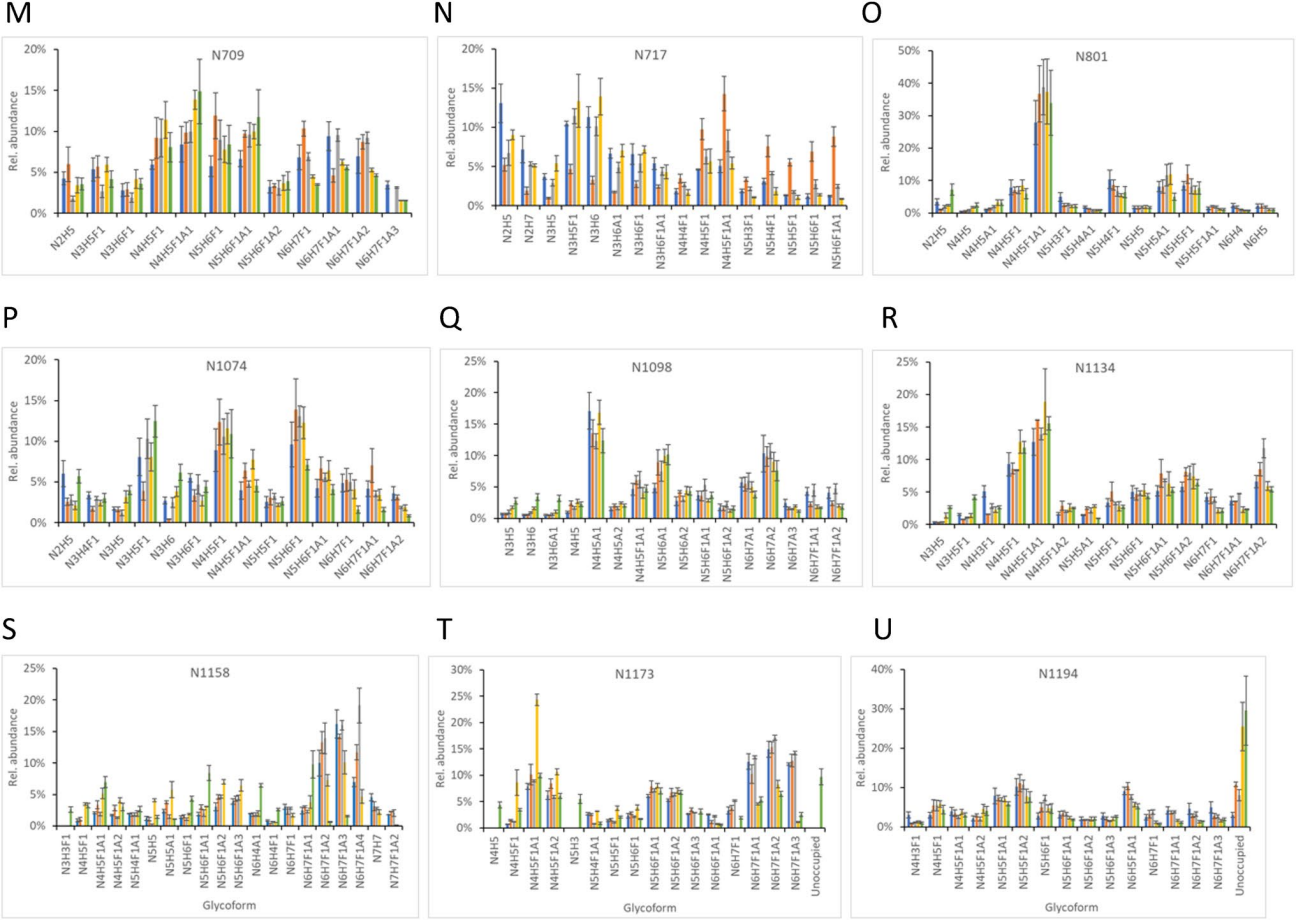
**A**–**U** Comparison of the relative abundance of the top ten (or more) N-glycans on each N-glycosylation sequon in an ectodomain of S proteins of five SARS-CoV-2 variants. D614G (blue), Alpha (orange), Beta (gray), Gamma (yellow), Delta (green). The results shown are the average of three analytical replicates with error bars representing the standard deviations

To evaluate and compare the microheterogeneous distribution of N-glycans attached to sequons, the relative abundance of at least ten most abundant glycans on a site of each S variant is shown in Fig. 2. Each bar graph was created by first selecting the top 10 glycans from each S variant, and then including lower-ranking glycans if the top 10 did not fully overlap between two or more variants. The smaller number of distinct glycans in the bar graphs indicates the higher overlap of the high abundant glycoforms among the five variants. For example, the N709 site was occupied dominantly by 12 glycans in all S variants (Fig. 2M), while the N1158 was mainly occupied by 20 shared glycans (Fig. 2S) showing a more diversified composition.

The diversity in N-glycan composition on specific glycosites of the S proteins was demonstrated by different patterns of relative abundances of distinct N-glycans. Similar to the N-glycan processing states, most of the sequons, including N17, N61, N74, N234, N282, N331, N343, N603, N616, N657, N709, N801, N1074, N1098, N1134, and N1194 (Fig. 2A, B, C, F, G, H, I, J, K, L, M, O, P, Q, R, and U, respectively), showed highly similar glycan distribution patterns in terms of glycoforms and their relative abundances among all S variants. However, other sequons (N122, N165, N717, N1158, and N1173) exhibited different patterns, with the abundance of some glycans differing between two or more spike variants. In the following abbreviated results, N is HexNAc, H is hexose, A is sialic acid, and F is fucose with numbers indicating stoichiometry. Compared to other variants, N122 of Delta S showed higher abundance for relatively low molecular weight glycans (N2H5, N3H5, N3H6, N4H5, N4H5A1) and lower abundance for higher molecular weight oligosaccharide groups (N4H5F1A1, N5H4F1A1, N5H5F1, et al.) (Fig. 2D). For N165, there were more glycoforms of N3H4, N4H5F1, and N5H5 in Delta S than in the other variants, and significant differences in other glycoforms were also observed (Fig. 2E). N717 of Alpha S displayed lower amounts of some relative low-density glycoforms but higher amounts of relatively large glycans than in other variants (Fig. 2N). N1158 and N1173 also showed changes in the abundance of some glycoforms in different S variants (Fig. 2S, T).

Amino acid substitutions on spike protein variants may affect their glycosylation and therefore affect the virus’s ability to infect host cells and evade immune responses. N165 is located near the receptor binding site on the SARS-CoV-2 S protein and the glycosylation of this site appears to play an important role in receptor binding and epitope shielding [5, 8, 61]. It has been shown by MD simulations that N-glycans at N165 directly interact with the residues of RBD to stabilize its open “up” structure and control the transition of the spike protein from open to closed conformation [25, 26]. Mutagenesis analysis has also demonstrated that removing glycans by mutating N165 to other amino acid residues significantly alters the binding of spike to ACE2, or neutralizing antibodies [21, 24, 25, 62]. Some studies have also showed that deleted glycosylation at N122, N717, and N1158 by mutations caused significant reduction in viral infectivity [21, 63, 64]. We observed in the present study that the glycosylation patterns of these sites varied between different VOCs, suggesting their potential role in enhanced virus transmission and infectivity. Further studies are needed to fully understand the molecular mechanism. Another difference in observed N-glycosylation patterns included some sequons occupied by many low abundance N-glycans, while other sequons were dominated by one or a few similar N-glycans in the five S variants. Sites with a limited number of glycan forms that represent summed abundances of 50% or more include N61 (Fig. 2B, N2H5, > 50%), N234 (Fig. 2F, N2H5, N2H6, N2H7, and N2H8; > 69%), N331 (Fig. 2H, N4H5F1, N4H5F1A1, N5H4F1, and N5H5F1, > 51%), N343 (Fig. 2I, N4H5F1, N4H5F1A1, N5H5F1, and N5H6F1, > 54%), N603 (Fig. 2 J, N4H5F1, N4H5F1A1, N5H5F1, and N5H6F1, > 47%), and N801 (Fig. 2O, N4H5F1, N4H5F1A1, N5H4F1, N5H5A1, and N5H5F1, > 59%). This is consistent with previously reported results that some sequons are less heterogeneous in N-glycans in the wild type and S variants [23, 24, 37, 51]. Two complex biantennary and monofucosylated glycans with or without a terminal sialic acid group, N4H5F1 and N4H5F1A1, were either separately or together the predominant glycans at many N-glycosites on some or all tested S variants, including N122, N165, N282, N343, N603, N616, N657, N709, N717, N801, N1074, N1134, and N1173 (Fig. 2). These results are also in agreement with the glycomic analysis reported by Shajahan et al. that the glycans of N4H5F1, N4H5F1A1, and N2H5 are the three most abundant glycoforms in the wild-type, Alpha, Beta, Gamma, and Delta variants of the S protein [56]. This might reflect the favorable local spatial compactness in the proteins and accessibility for the formation of these specific glycans.

**Fig. 3:**
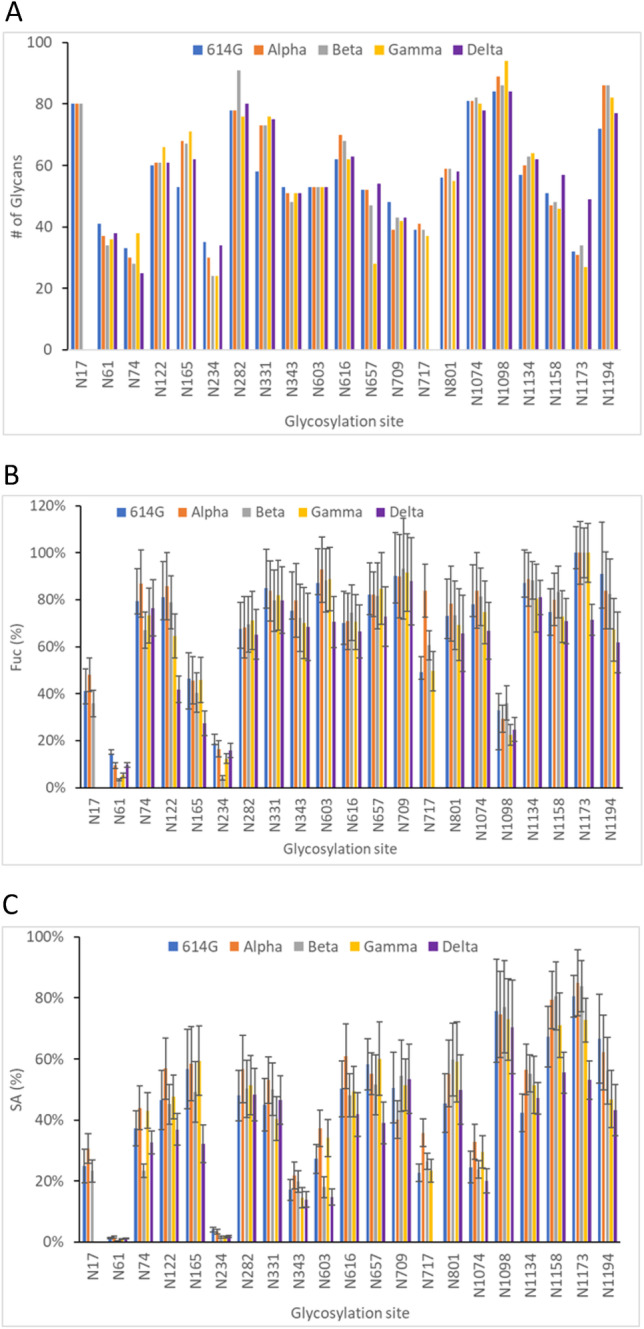
The number of detected N-glycans at 21 conventional sequons (**A**) and the relative abundance of fucosylated (Fuc) (**B**) and sialylated (SA) glycans (**C**)

Microheterogeneity is a common feature of N-glycosylation [65]. The number of distinct N-linked glycans at each sequon on the spike variants tested varied ranging from 24 to 91 (Fig. 3). The total number of the glycans at any single N-glycosylation site remained the same or in similar numbers across the variants, with a few exceptions such as N182 for Beta, N657 for Gamma, and N1173 for Delta (Fig. 3A). Some sites such as N61, N74, N234, N717, and N1173 exhibited relatively low heterogeneity with less than 40 glycoforms while others such as N17, N282, N1074, N1098, and N1194 contained approximately 80 different glycans. The low number of glycans at N61 and N234 should be related to the limited number of oligomannose glycoforms (H2N5 to N2N9) present mainly at these sites while the cases of N74, N717, and N1173 might be due to a restricted sequon accessibility by the enzymes required to process oligosaccharides [51]. Taken together, the strong similarities in glycan types, forms, and numbers of unique glycan identities across five S variants suggested mutation/deletion of amino acids in these variant proteins did not significantly affect their overall N-glycosylation profiles or presumably their global conformations and total glycan shielding.

Many N-glycans contain sialic acid and fucose groups. These sialylated and fucosylated glycoforms implicated a range of immune system diseases [18, 65, 66]. Similar trends were observed on glycan fucosylation and sialylation on most of the N-glycan sites while the abundance of fucosylated and sialylated glycans varied at several sequons across all the variants. The level of sialylation and fucosylation of the glycans at two specific sequons, N61 and N234, were particularly low (5% and 20%, respectively), due to the high abundance of oligomannose glycans without fucose and sialic acid groups attached. In addition, N1098 possessed more sialylated glycans (70.5–77.1%) than fucosylated ones (22.4–36.0%). In contrast, the sites of N343 and N603 contained a higher percentage of fucosylation (68.4–79.6% and 70.5–92.8%, respectively) than sialylation (14.0–21.9% and 14.7–34.2%, respectively). It has been revealed in a recently published paper that the sialic acid and fucose content in SARS-CoV-2 RBD affects the reactivity of antibodies in serum samples from COVID-19 patients [67]. They found that there is more fucosylated and less sialylated complex glycans present in HEK-expressed RBD proteins, and that removal of only sialic acids or terminal fucoses enhances antibody reactivity while removal of all glycans diminishes antibody reactivity. We also observed high abundance of fucosylation and low abundance of sialylation on two sequons, N343 and N331, of the tested S variants. The difference in the degree of fucosylation and sialylation might alter N-glycan shielding properties of these S variants, ultimately affecting immune evasion and virus infectivity.

### N-glycosylation on two novel sequons in Gamma variant

**Fig. 4:**
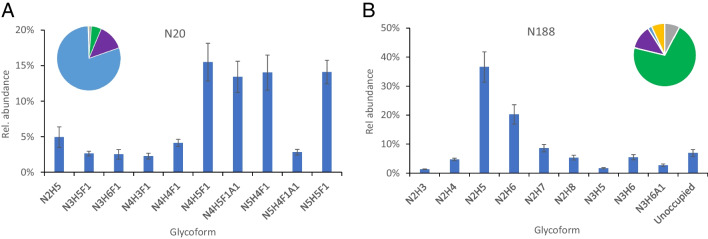
N-glycan profiles of two novel sequons including N20 (**A**) and N188 (**B**) introduced by mutations in the Gamma variant and the percentage distribution of glycan types (inserts). The colors in the pie charts represent the same glycan types as shown in Fig. 1

N-glycosylation sequons include a tri-residue consensus sequence, NX(S/T). Some point mutations of viral glycoproteins can alter their glycosylation profile by generating new sequons on the antigens [68]. Compared to the SARS-CoV-2 wild-type and other variant S, the Gamma S has two novel NXT/S N-glycosylation sequons (Table 1). The mutation T20N converts the T_20_R_21_T_22_ in other S sequences into N_20_R_21_T_22_ in Gamma, generating a potential N-glycosite at N20. Likewise, the R190S mutation in Gamma leads to the formation of another sequon, N_188_L_189_S_190_, and thus N188 becomes a potential N-glycosylation site. In contrast, the T19R mutation changes the sequon of N_17_L_18_T_19_ into N_17_L_18_R_19_ in Delta spike, removing its N17 glycosylation site. Our LC–MS/MS data revealed that glycopeptides containing these two novel Gamma sequons (N20 and N188) were detected and the sites were almost fully occupied by various N-glycans. Figure 4 shows the percentage of N-glycan types and the distribution of high abundance N-glycan forms. Approximately 80% of the N20 site peptides located at the N-terminal region of the N-terminal domain (NTD) were modified with fully processed complex glycans at high level of fucosylation (92%) and sialylation (29%). The other novel site N188, however, was mainly oligomannose glycans (71%) and the site contained low abundances of fucosylated and sialylated glycans. These findings are consistent with the results reported by Newby et al. [55].

N20 and N188 are surface-exposed residues in the NTD of the Gamma S protein [15, 69]. In addition to RBD, the NTD of SARS-CoV-2 S has been found to contain antibody binding sites (so-called “NTD supersites”) in the region lined by N17, N74, N122, and N149 glycans [70–72] and neutralizing antibodies, for example, 4A8, target NTD residues and inhibit changes in RBD conformations presumably through allosteric interactions [73]*.* A recent study has revealed that the hyperglycosylated S of Gamma seems to be more stable than the S protein of wild-type, Alpha, and Beta variants [74]. The addition of N-glycans to N20 and N188 residues might facilitate epitope shielding and protein stability. The disappearance of these two sequons in the next circulating variant, Delta, implies that N-glycosylation at these two sites might have been replaced by a more protective structural alteration.

## Conclusion

In this report, we applied an advanced LC–MS/MS method to characterize the N-glycosylation profiles of four SARS-CoV-2 Variant of Concern spike proteins including Alpha, Beta, Gamma, and Delta along with D614G as a control sample. To avoid the inherent limitations in the LC–MS/MS analysis of glycopeptides and to provide accurate comparison, we used recombinant proteins obtained from one manufacturer and applied multiple protease digestion strategies. Additionally, only the glycosylation data with consistent results from at least two different digestion experiments were considered. We identified N-glycans and quantified their relative abundances at most sequons including two novel sites of Gamma S. Our results revealed that the amino acid substitutions and deletions between variants had some impacts on the abundance and type of glycans on glycosylation sites of the S protein. Some N-glycosylation sequons in S showed a difference between these variants in the distribution of various glycan forms (oligomannose, hybrid, and complex). On the other hand, we observed high similarity in the N-glycosylation of the SARS-CoV-2 S among all five variants examined in terms of glycoforms, occupancy, process state, numbers of glycans, fucosylation, and sialylation. This suggests that the mutations in these variants did not significantly alter the distribution of their N-glycosylation and these modifications still maintain their major role supporting overall protein stability and efficient immune shielding. The observed variations on some NTD residues, such as N74, N122, and N165, might cause changes in the interaction of S and ACE2 and further investigation is required to understand the underlying mechanism(s). Taken together, the highly similar N-glycosylation of spike proteins from different SARS-CoV-2 variants suggests that this modification is conserved across these lineages of the virus. This could indicate that N-glycosylation is critical for the proper structure and function of the spike protein, and that changes in these modifications could result in significant alterations to the virus. This fact also suggests that existing vaccines may continue to be effective against new variants. However, further research is needed to confirm this, as there may be differences in the precise composition of N-glycans among different variants that could impact vaccine efficacy.

## Limitations of the study

We applied a multiple enzyme digestion method and a new strategy to select the best dataset for a glycosylation site when at least two digestion experiments yielded similar results. However, this approach might result in the omission of detection of glycosylation profile at some potential sites, for example, N149. More experiments with varied proteases and other experimental conditions should be explored to detect and quantify glycopeptides on all potential N-glycosylation sites, and also to characterize O-glycosylation, sulfulated, and phosphorylated glycans. Another limitation of this study is that the structural features of glycoforms such as glycan linkages, LacdiNAc and mannose 6-phosphate et al., could not be determined. The presence of the less-common LacdiNAc moiety in the glycans of HEK expressed glycoproteins might lead to over-interpretation of complex type glycan abundance. This might be resolved by adjusting the collision energy to break specific glycan linkages on glycopeptides during mass spectrometry data acquisition, and by applying specific algorithms [47, 75, 76]. Other factors that might cause bias include source of the recombinant proteins, parameters in MS acquisition, and data processing program [57]. For instance, different lots of recombinant proteins might have different glycosylation patterns even the proteins were obtained from one manufacturer. We used the same lot of materials for each protein in this study that might cause certain degree of bias.

## Supplementary information

Below is the link to the electronic supplementary material.

Supplementary file1 (XLSX 241 kb)

Supplementary file2 (DOCX 25 kb)

## Data Availability

Raw LC–MS/MS data have been deposited on the Mass Spectrometry Interactive Virtual Environment (MassIVE) repository server. The accession ID is MSV000091492.
