# Supplementary material for: Analysis of the N-glycosylation profiles of the spike proteins from the Alpha, Beta, Gamma, and Delta variants of SARS-CoV-2
Source: Anal Bioanal Chem. 2023 Jun 24;415(19):4779–93. doi: 10.1007/s00216-023-04771-y (PMC10352417; doi:10.1007/s00216-023-04771-y)
Supplement: Supplementary file 2 — Supplementary file2 (DOCX 25 kb) [file 216_2023_4771_MOESM2_ESM.docx]

Supplementary information

**Analysis of the N-glycosylation profiles of the spike proteins from the Alpha, Beta, Gamma, and Delta variants of SARS-CoV-2**

Dongxia Wang*, Jakub Baudys, Sarah H. Osman, John R. Barr*

National Center for Environmental Health, Division of Laboratory Sciences. Georgia, United States of America.

*To whom correspondence should be addressed.

Dongxia Wang, Ph.D.: telephone 770-488-0446, email [dov2@cdc.gov](mailto:dov2@cdc.gov), fax 770-488-0509

John R. Barr, Ph.D.: telephone 770-488-7848, e-mail [jbarr@cdc.gov](mailto:jbarr@cdc.gov), fax 770-488-0509.
